# Live Cell Interferometry Quantifies Dynamics of Biomass Partitioning during Cytokinesis

**DOI:** 10.1371/journal.pone.0115726

**Published:** 2014-12-22

**Authors:** Thomas A. Zangle, Michael A. Teitell, Jason Reed

**Affiliations:** 1 Department of Bioengineering, University of California Los Angeles (UCLA), Los Angeles, California, United States of America; 2 Jonsson Comprehensive Cancer Center, UCLA, Los Angeles, California, United States of America; 3 Department of Pathology and Laboratory Medicine, David Geffen School of Medicine at UCLA, Los Angeles, California, United States of America; 4 California NanoSystems Institute, UCLA, Los Angeles, California, United States of America; 5 Broad Stem Cell Research Center, UCLA, Los Angeles, California, United States of America; 6 Molecular Biology Institute, UCLA, Los Angeles, California, United States of America; 7 Department of Physics, Virginia Commonwealth University (VCU), Richmond, Virginia, United States of America; 8 VCU Massey Cancer Center, Richmond, Virginia, United States of America; University of Turin, Italy

## Abstract

The equal partitioning of cell mass between daughters is the usual and expected outcome of cytokinesis for self-renewing cells. However, most studies of partitioning during cell division have focused on daughter cell shape symmetry or segregation of chromosomes. Here, we use live cell interferometry (LCI) to quantify the partitioning of daughter cell mass during and following cytokinesis. We use adherent and non-adherent mouse fibroblast and mouse and human lymphocyte cell lines as models and show that, on average, mass asymmetries present at the time of cleavage furrow formation persist through cytokinesis. The addition of multiple cytoskeleton-disrupting agents leads to increased asymmetry in mass partitioning which suggests the absence of active mass partitioning mechanisms after cleavage furrow positioning.

## Introduction

The partitioning of cell contents during division is fundamental for growth and development of metazoans [1], [2]. Most work on the partitioning of cellular contents during the division of cells in homeostasis has focused on either the partitioning of genetic material or on cell shape regulation. In these studies, condensed chromosome segregation can be assessed by either direct visualization of dense, highly refractive chromosomes or by semi-quantitative microscopy with fluorescent nucleic acid stains [3]. This work suggests that chromosome segregation is actively regulated during cell division. Cell shape during and following division can be visualized and scored using common fluorescence techniques, and also appears to be actively regulated during cell division [4]. By contrast, much less is known about the partitioning of non-genetic cellular constituents during and after division, although non-genetic elements may have large roles in regulating cell fate and function [5], [6]. For example, in *C. elegans* partitioning of the PAR proteins have been shown to determine cell polarity during growth and development [7], [8].

Prior work on the partitioning of non-genetic material after cytokinesis has shown a moderate degree of asymmetry in cultured cell lines, which are typically assumed to divide their contents symmetrically. Fuentealba *et al.* [9] showed that many cell divisions in culture unequally partition proteins targeted to the proteasome. In human multiple myeloma H929 cells, Reed *et al.* [10] showed that some cell divisions result in daughter cells of unequal biomass. Tzur *et al.* [11] measured volume asymmetry and Sung *et al.* [12] measured biomass asymmetry at division and found a moderately high (8–10%) average mass asymmetry which points to an active mechanism for cell size regulation. This result contrasts with experiments that suggest mammalian cell growth is independent of cell size [13]. Furthermore, Reed *et al.* [10] showed that for some cell divisions, cell mass is dynamically redistributed between daughter cells during cytokinesis before complete daughter cell separation. It remains unclear whether mass partitioning between daughter cells during cytokinesis is an actively regulated process, or if the primary regulation of cell size occurs later in the cell cycle, as has been suggested by recent work on growth dynamics during the cell cycle [14].

Cell division is a mechanical process linked to the cytoskeleton. Elucidation of the temporal mechanisms regulating force generation should yield insights into the processes underlying cell division [15]. At any given time in the human body 10^8^–10^9^ divisions occur, so mechanisms that monitor and regulate cell shape progression during cytokinesis are essential because there are no checkpoints preventing excessive growth if cytokinesis fails [16]. Therefore, it has been hypothesized that there must be a mechanosensory system that monitors cell shape during division [16]. Evolutionarily, it has been suggested that the earliest steps in regulating cell division may have been entirely mechanical and decoupled cells from the osmolarity of the environment and Myosin II activity [17]. However, recent data shows that mammalian cell structure during division is not decoupled from osmolarity and is highly dependent on cytoskeletal activity, particularly in the cell cortex [18]. Additional work demonstrates that the interaction between cytoskeletal components and cellular motors is essential for regulating cell shape during division. In studies of the slime mold *Dictyostelium discoideum*, it was shown that asymmetries in cell shape from a distended cell cortex during cytokinesis can be corrected through increased concentrations of Myosin II at the site of the perturbation. This mechanical adjustment locally increases cortical tension, increasing shape symmetry between daughter cells [19]. Other recent work on mammalian cells has shown that membrane blebbing can help move lipids during cytokinesis to increase the symmetry of initially asymmetric divisions, in addition to the function of microtubules in initial centering of the actin ring prior to cytokinesis [4].

While previous studies of cell division have focused on active regulation of chromosome segregation and daughter cell shape during division, here we use live cell interferometry (LCI) to quantify the temporal dynamics of biomass partitioning during cell division. LCI measures the phase shift of light as it passes though and interacts with biomass inside of cells to precisely determine the dry mass of single cells [10], [20]. LCI biomass measurements are independent of cell shape, allowing precise measurements of the partitioning of cellular contents during and immediately following cell division. We also investigate the role of cytoskeletal disruption on mass partitioning fidelity.

## Results

### LCI tracks cell mass partitioning during division

We evaluated unsynchronized L-cells growing in culture using LCI [10]. Each cell was automatically tracked over time, to yield traces of mass, shape factor and mean phase shift (which is linearly proportional to the average projected mass per area [10], [21], [22]). The mean phase shift rises sharply just prior to cell division and falls as the adherent L-cells reattach to the substrate. Cell divisions were automatically detected by identifying mass traces that met three criteria: 1) The presence of two daughter cells in the immediate vicinity of the putative parent cell with each daughter having roughly 50% of the parent cell mass; 2) A good fit of the characteristic increase in mean phase shift versus time plot of the pre-division parent cell with a sigmoidal-shaped filter; and 3) A good fit of the characteristic decrease in mean phase shift versus time plot of the two daughter cells post-division to a sigmoidal filter (S1B Fig (inset)). Daughter cell tracks are defined as starting in the image in which the two cells are separated by the watershed transform, which corresponds to the presence of a deep cleavage furrow and the start of cytokinesis. The masses of individual daughter cell pairs were tracked from this point onwards, which includes a period where the two daughter cells are substantially connected to one another.

**Figure 1:**
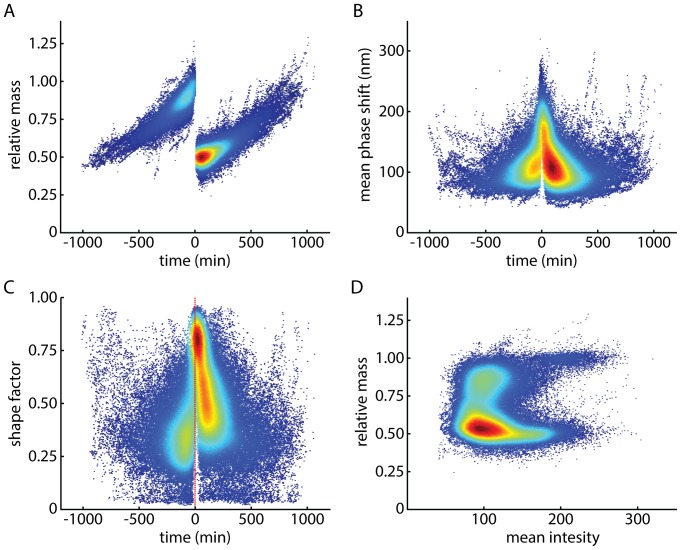
Mass, mean phase shift, and shape factor traces for untreated L-cell divisions. (A) Mass of dividing L-cells relative to parent cell mass. *t* = 0 corresponds to the last parent cell mass measurement, just prior to cleavage furrow formation. Color corresponds to the number of observed mass measurements at a given time and mass coordinate, with red being highest number of measurements and blue being lowest. (B) Mean phase shift versus imaging time. (C) Shape factor, defined as 4π*A*/*P* ^2^, versus imaging time. (D) Relative mass versus mean phase shift. Mitotic cells are in the upper right quadrant, recently divided cells in the lower right quadrant.

Cell biomass versus imaging time for 542 dividing L-cells was plotted, with mass normalized to the mass of the parent cell at the time of deep cleavage furrow formation, *t* = 0 (Fig. 1A). Note that the density of traces is highest close to the time of division. This is because individual traces were normalized to the mass of the parent cell at *t* = 0, so the data is expected to collapse at this point. Also, the only traces shown are those for which successful divisions were detected, and most traces do not last for a complete cell cycle, due mainly to tracks being lost when cells move over one another as is common in adherent cell culture. The data show an increasing mass accumulation rate with time over the course of the cell cycle (S2 A-C Fig.), which is generally consistent with previous cell-cycle linked mass accumulation rates in mammalian cells [14], [23]. We note, however, that there have been a variety of specific mass accumulation rate behaviors reported, showing variously, an abrupt increase in growth rate at the end of the cell cycle [23], or more finely regulated growth control [14]. The average cell dry mass (405 pg) is within the range reported for various mammalian cells (U2OS: 50–100 pg [23], H929: 200–400 pg [10], CD8, M202: 75–800 pg [24]). Mean phase shift of the phase signal (Fig. 1B) and shape factor (Fig. 1C) both show an abrupt increase just prior to, and immediately following cell division due to cell rounding during mitosis and subsequent reattachment to the substrate following cell division. A plot of relative mass versus mean phase shift (Fig. 1D) shows progression through the cell cycle, with newly divided cells, at *m* = 0.5 *m_parent_* and high mean phase shift (lower right quadrant), cells attached to the substrate moving from the lower left quadrant to the upper left quadrant through the cell cycle, and cells just about to divide in the upper right quadrant.

**Figure 2:**
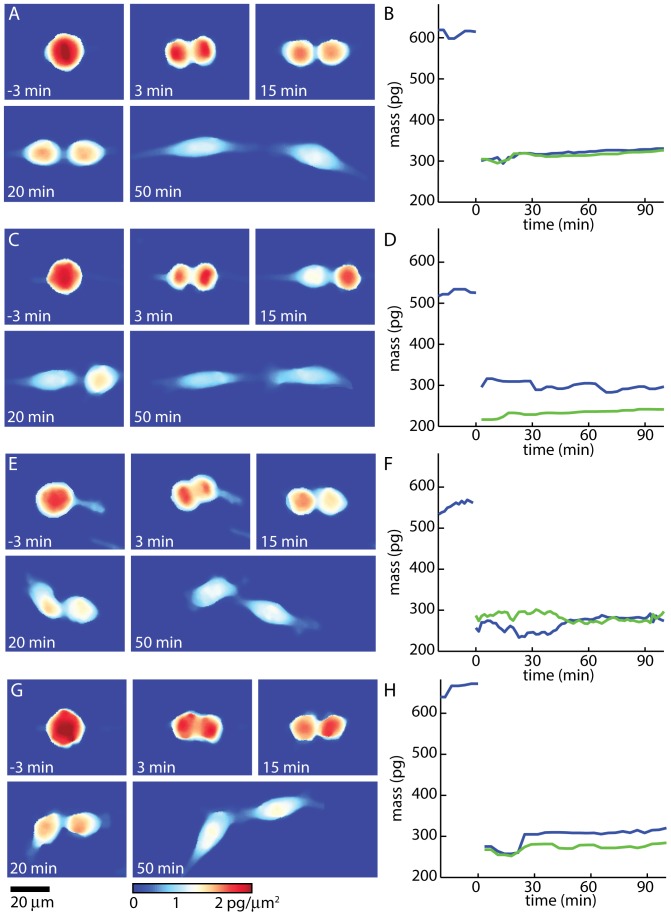
LCI tracks daughter cell mass distributions during division. (A, C, E, G) Mass distribution during three representative mouse L cell divisions. (B, D, E, H) Mass versus time for three mouse L cell divisions. A, B, show a symmetric division. C, D show an asymmetric division. E and F show a division where, after cleavage furrow ingression (*t* = 0 min), mass is initially asymmetrically partitioned between the two daughter cells. By the time the two daughter cells visibly separate (*t* = 50 min), the mass of the two daughters is approximately equal. G, H show a division which starts out symmetric, and ends with an asymmetric partitioning of mass. Colormap and scalebar below G are the same for all images in A, C, E, and G. A video of individual cell divisions is provided in S1-S4 Movies.

Based on a sigmoidal fit to the average mean phase shift versus imaging time data (Fig. 1B), L-cells spend an average of 26 minutes rounded up post-division prior to flattening onto the substrate and 32 minutes rounded up pre-division (S2D Fig.). Therefore, we used LCI to observe mass partitioning between daughter cells during this crucial period of late cytokinesis, after the formation of a deep cleavage furrow but before cells separate and reattach to the substrate. During this period, a range of mass partitioning behaviors was observed (Fig. 2). In contrast to earlier work which only observed a handful of cell divisions and only reported a limited range of mass partitioning behaviors [10], these results show a wide range of mass partitioning behaviors during late cytokinesis, from purely symmetric (Fig. 2 A,B) or asymmetric (Fig. 2 C,D) divisions, to divisions which show a redistribution of mass, which either reduces (Fig. 2 E,F) or increases (Fig. 2 G,H) the mass asymmetry between daughter cells.

### Mass partitioning dynamics during cell division

**Figure 3:**
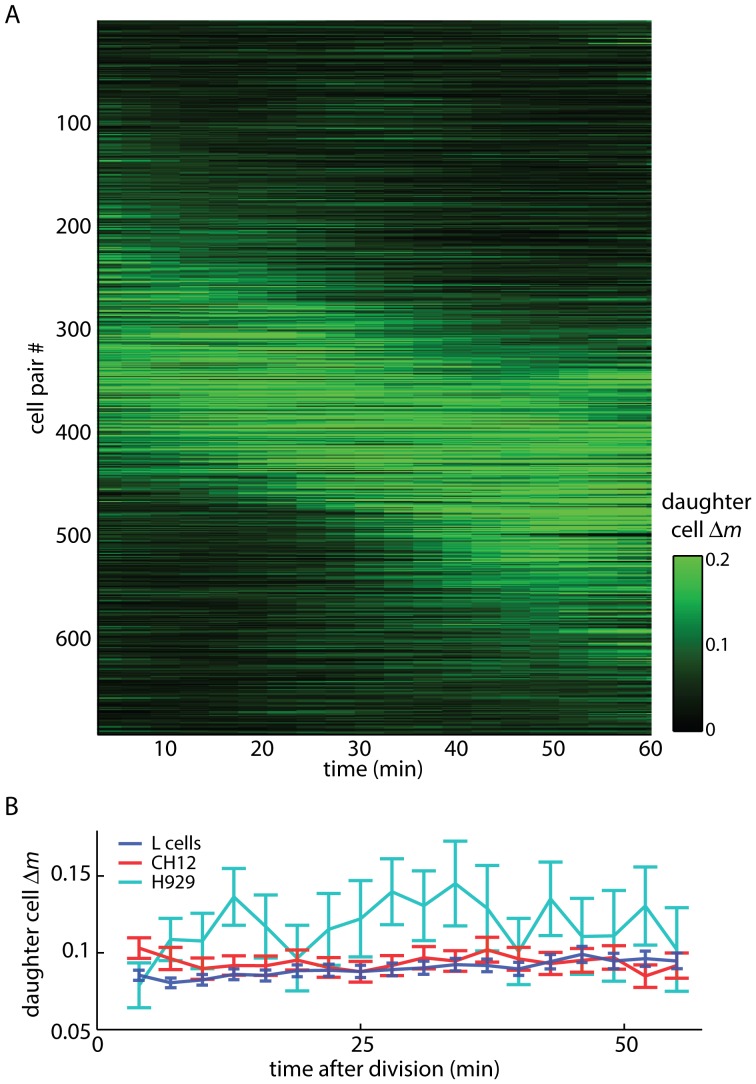
Daughter cell mass difference versus time after division. (A) daughter cell mass versus time for 691 control (untreated and DMSO treated) mouse L cell divisions, ordered by the slope and intercept of daughter cell Δ*m* show a range of mass partitioning behaviors (symmetric, asymmetric, redistributing). (B) Average mass partitioning asymmetry (absolute value of daughter cell Δ*m*) versus time for three cell lines shows little change in the average Δ*m* over time. Error bars represent s.e.m. of all tracked divisions. *n* = 437 untreated L cells, *n* = 197 CH12 cells, *n* = 19 H929 cells.

We visualized L-cell the mass asymmetry between daughter cells as a heatmap (S1C−D Fig.) to show behavior of the entire population (Fig. 3A), and the relative proportion of cells displaying each general class of mass redistribution behavior. In this plot, black indicates low asymmetry, with green corresponding to high absolute asymmetry between daughter cells. To order the heatmaps of daughter cell mass asymmetry, we first performed a linear least squares fit to the division asymmetry versus time, to get the rate, *r*, at which daughter cell mass differences were increasing or decreasing. Ordering solely based on this rate would cluster cell divisions with low slope but large (asymmetric) or small (symmetric) mass partitioning asymmetries (Δ*m_0_*) towards the center of the plot. To map both the rate of mass partitioning asymmetry increase/decrease and the magnitude of the initial asymmetry to a single dimension (the cell pair # axis in Fig. 3A), we ordered the heatmaps using the four-quadrant inverse tangent of the slope of the asymmetry versus time over the initial cell mass partitioning asymmetry: atan2(*r*,Δ*m_0_*). This ordering separates cell mass partitioning behaviors into roughly four groups from top to bottom: initially symmetric with little change in asymmetry; initially asymmetric with a decrease in asymmetry; initially asymmetric with little change in asymmetry; and initially symmetric with an increase in asymmetry.

We also tracked mass partitioning of non-adherent H929 and CH12 cell divisions. The average mass asymmetry relative to the average daughter cell mass for all cell lines is ∼10%, which is consistent with adherent L-cell data as well as previous results for a variety of mammalian cell lines [11], [12]. On average, initial mass partitioning asymmetries persist through the completion of cell division, and the daughter cell that is larger initially ends up larger than its sister (Fig. 3B), consistent with the absence of mass partitioning checkpoints acting during cytokinesis. This mass partitioning asymmetry shows no dependence on cell confluence over the range of 5–35% that was experimentally accessible (S3A and B Fig.) or on total time in culture (S3C Fig.). The observed 10% asymmetry between daughter cells is smaller than both the baseline, cell cycle synchronized size variation among all cells in the population (14%), and the 20% volumetric size variation among unrelated newborn cells reported in Tzur *et al.* [11].

### Cytoskeletal disruptions give rise to an increase in division asymmetry

Data suggests a mechanosensory system linking cell shape to the action of motor proteins during cytokinesis [25]. To determine the influence of cytoskeletal disruption on mass partitioning during and following cytokinesis, mouse L-cells were treated with a panel of cytoskeletal disrupting agents at concentrations sufficient to perturb but not completely block cell division. The agents chosen include latrunculin A (100 nM), which binds Actin monomers, blebbistatin (10 µM), which inhibits Myosin II activity, nocadozole (75 nM) which inhibits Microtubule polymerization, and cytochalasin B (2 µM), which inhibits Actin polymerization [26]. Each drug was added 1 h before the start of imaging. Cells were imaged for 20 h, to capture the first cell divisions after treatment began.

Drug concentrations were determined in a series of calibration experiments, starting at target concentrations based on prior work examining cell rounding pressure during mitosis [18] and increasing or decreasing the drug concentration by factors of two (S4 Fig.). The final treatment concentration was equal to the highest concentration at which cells successfully completed cytokinesis at a rate sufficient to track approximately 30 divisions per experiment (S4A Fig.), to ensure adequate statistical power of mass partitioning results. In addition to successfully completing cytokinesis, cells at the target drug concentrations showed positive growth rates (S4B Fig.), suggesting that the concentrations chosen for analysis are neither cytostatic nor cytotoxic. The difference in growth rate at most conditions relative to the DMSO control was not statistically significant, aside from the mid and high concentrations of nocodazole, which showed a slight decrease (S4B Fig.). The effects of these drugs on initial mass partitioning asymmetry did not vary significantly over the 20 h treatment period (S4C Fig.), suggesting that the drugs remained effective for the duration of the treatment and imaging period.

**Figure 4:**
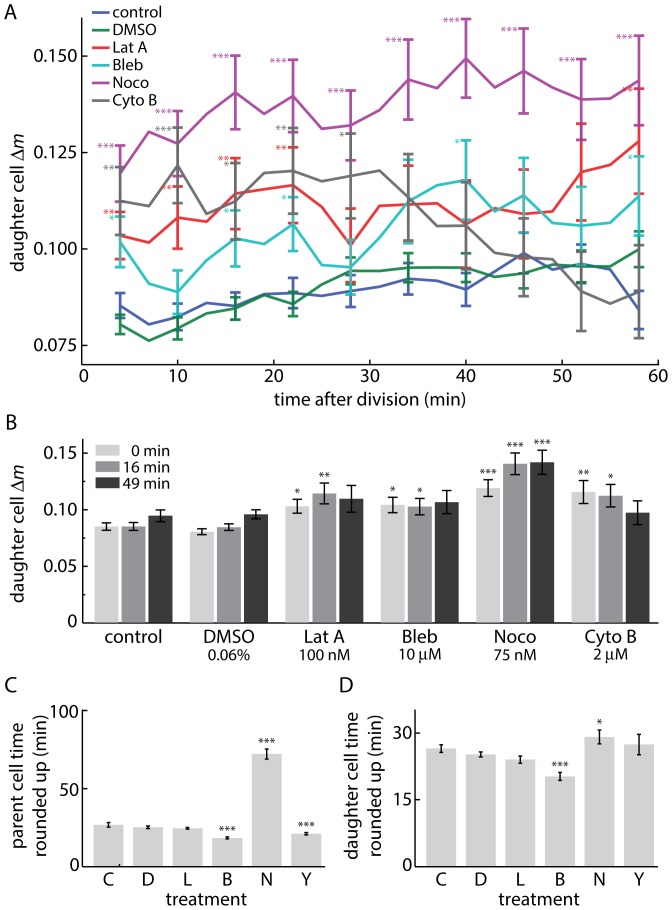
Daughter cell mass partitioning results with cytoskeletal disruption. (A) Absolute value of daughter cell Δ*m* show an increase in mass partitioning error with cytoskeletal disruption. (B) Daughter cell Δ*m* at select (approximate) time points from A. (C) time parent spent rounded up in mitosis prior to division. (D) time daughters cell spent rounded up before first daughter cell flattened out. C: control (*n* = 437), D: DMSO 0.06% (*n* = 587), L: latrunculin A 100 nM (*n* = 132), B: blebbistatin 10 µM (*n* = 129), N: nocodazole 75 nM (*n* = 116), Y: cytochalasin B 2 µM (*n* = 81). Error bars represent s.e.m of all tracked divisions. * *p*<0.05, ** *p*<0.01, *** *p*<0.001. Statistical significance in A and B was determined relative to control measurements at each corresponding timepoint.

Disruption of specific cytoskeletal components crucial for spindle positioning and maintenance of cell shape increases division asymmetry (Fig. 4A−B). The temporal dynamics of this effect varies depending on the specific mode of inhibition. Microtubule function is essential for spindle positioning prior to cytokinesis [4], [27]. In cells which successfully complete cytokinesis after treatment with sub-cytoxic concentrations of nocadozole, mass partitioning between daughter cells is highly asymmetric, and cells take a long time to exit mitosis (Fig. 4C). This is consistent with previous work showing that interfering with MT dynamics leads to chronic activation of the spindle assembly checkpoint, leading to prolonged mitotic arrest [28].

Additionally, although latrunculin A and blebbistatin have previously been shown to have opposing effects on cell rounding pressure [18], they have very similar, moderate effects on cell mass asymmetry. This is consistent with previous work, which showed that Actomyosin activity is required to maintain the position of the cleavage furrow [29]. Cytochalasin B, which blocks the formation of new actin filaments, causes a transient increase in daughter cell asymmetry which decreases back to control levels over time (Fig. 4A) as cells separate from one another, reattach to the substrate (Fig. 4D) and become less rounded up following separation (S5 Fig.).

## Materials and Methods

### Cell Lines

Mouse L-cell fibroblasts and CH12 B cells, along with H929 human myeloma cells were acquired from the American Type Culture Collection (ATCC). L-cells were cultured in DMEM supplemented with 10% fetal bovine serum (FBS, Omega Scientific), 1x non-essential amino acid solution (Invitrogen), 2 mM glutamine (Invitrogen) and antibiotics. CH12 cells were cultured in RPMI 1640 supplemented with 10% knock-out FBS, 5% NCTC-109 media (Invitrogen), 71 µM β-mercaptoethanol (Sigma), 1 mM sodium pyruvate (Invitrogen), 1x non-essential amino acid solution, 2 mM glutamine and antibiotics. H929 cells were cultured in RPMI 1640 supplemented with 10% defined FBS (HyClone) and antibiotics. L-cells were switched to phenol-red free DMEM 24 hours before plating to reduce phototoxicity.

### Drug treatments

Blebbistatin (Tocris Bioscience), latrunculin A (Sigma), cytochalasin B (Sigma), and nocodazole (Sigma) were suspended in DMSO at the manufacturer's recommended concentrations and added to cell culture media just prior to the addition of cells to the microscope stage, 1 hour before the start of imaging. DMSO carrier control experiments (*n* = 734 tracked divisions) were performed at a DMSO concentration of 0.06%, representing the highest concentration of DMSO used for drug treatments. A series of dose-response experiments were performed to determine drug concentrations at which cell division was impeded, but a substantial fraction of cells were still able to complete cytokinesis (S4A Fig.). In these experiments, drug concentrations were increased until there was a significant decrease in the number of cell divisions that were tracked in a given experimental location, from approximately 0.15 successful cell divisions per hour, per location, down to between 0.05 and 0.1 tracked divisions per hour, per location. This empirical rate of cell division (the exact value of which depends on cell confluence, which here was 5–35% (S3A Fig.), and field of view, which here was 240 by 320 µm) was sufficient to obtain a minimum of ∼80–100 tracked divisions in three, 20 h experimental trials. Therefore, this dosing was chosen as a practical balance between perturbing cell division and still allowing for statistical significance in the results. All experiments beyond the dose-response trials were performed at these final drug concentrations, which were: latrunculin A: 100 nM, blebbistatin: 10 µM, nocodazole: 75 nM, cytochalasin B: 2 µM.

### Live Cell Imaging

20 mm×20 mm silicon slides were treated with a solution of 70% ethanol in water, then dried and cleaned with deionized water, followed by cell plating at ∼8×10^4^ cells/slide. For non-adherent CH12 and H929 lymphoid cells, silicon slides were treated with a 0.01% solution of poly-l-lysine (Sigma) prior to plating. Plated cells were grown in a cell culture incubator for 24 h before the start of imaging. Silicon slides were placed into a custom-built fluid-recycling perfusion incubation system at 37°C, with built-in heaters, immersed temperature probe, and CO_2_/pH control via gas (5% CO_2_, 21% O_2_, balance N_2_) bubbled into the attached media reservoir [10], [24].

Cells were imaged continuously for 20 h using either a GT-X8 (Bruker) or NT9300 (Bruker) optical profiler modified to accommodate the custom live cell incubation system and a 20x, 0.28 numerical aperture microscope objective with attached Michelson interferometer. The Michelson interferometer consisted of a beam-splitter, reference mirror, and compensation reference chamber filled with deionized water. Illumination was provided by a 530 nm fiber-coupled LED (Thorlabs). In each experiment, 24–30 locations with sufficient spacing between cells to allow for automated image processing and mass segmentation were chosen and imaged at 3 min intervals.

### Phase Unwrapping

The first step in data analysis was to remove integer-wavelength errors in the initial calculation of phase shifts, a process called phase unwrapping which is common to all quantitative phase techniques [30]. We used an algorithm based on random-walker image segmentation [31] combined with an optimized set of image filters. Briefly, a training dataset was constructed by manually applying single-wavelength corrections to approximately 50 full images of phase data, selected for the representative appearance of a variety of adherent cell morphologies. Only single, positive wavelength corrections were applied, as multiple, or negative wavelength corrections were very rare, thereby reducing the problem to a two-region image classification problem. This training dataset (1.5×10^7^ pixels) was used in a linear discriminant analysis (LDA) to find the linear combination of 16 different pixel statistics (including the raw image, the intensity image, the phase quality magnitude image, and multiple edge-detection filters) that gives the best identification of which pixels lie on the boundaries of phase wrapped regions. The LDA coefficients applied to the 16 different pixel statistics gives an image which was then segmented using random-walker based image segmentation into regions that required correction, and those that did not, using a threshold score chosen based on the training dataset.

### Cell Mass Tracking

A custom Matlab (Mathworks) script was used to track the mass of individual parent and daughter cells. Phase corrected images were filtered with a Gaussian low-pass filter before image segmentation base on the watershed transform and Otsu thresholding (to determine local maxima corresponding to individual cells). This algorithm separates individual cells from their neighbors by identifying the ‘ridge’ in phase shift intensity between local phase shift maxima. Cell mass was estimated from phase shift measurements using a specific refractive index of 1.8×10^−4^ m^3^/kg, which represents a whole-cell average value [32]. Cell mass is computed as the sum of the mass per pixel over the segmented area of the cell. Cell tracking to link individual cell mass measurements over time was performed using a particle tracking code implemented in Matlab by Daniel Blair and Eric Dufresne, based on an algorithm originally applied to the study of colloidal particles [33]. Cell confluence was measured as the percentage of the total imaging area covered by cells.

Mitotic entry in adherent cells was determined by finding the time at which the mass per area of a cell increased abruptly as it detached from the substrate and rounded up. This was performed automatically in Matlab by pattern-matching the mean phase shift (which is linearly proportional to the mass per area) with a sigmoidal filter and looking for a peak greater than a user-defined threshold. The time of this peak corresponds to roughly the time at which mean phase shift has risen or decayed to 50% of its maximum value, corresponding to the midpoint in cell rounding/flattening. Cell division in adherent cells (L cells) was detected by a mitotic entry event followed by splitting into two daughter cells of roughly 50% of the mass of the parent cell which show an abrupt decrease in mean phase shift (mitotic exit). This corresponds to the parent cell rounding up and daughter cells flattening before and after division, respectively. Cell division in non-adherent cells (CH12, H929), which do not change shape substantially during cytokinesis, was detected by identifying a parent cell splitting into two daughter cells of ∼50% of the mass of the parent cell. The relatively consistent shape and area of non-adherent cells, with a single phase shift maxima located at the nucleus, resulted in fewer false segmentations via the marked watershed algorithm than with adherent cells, and so this extra step to verify that a division occurred was not necessary. Cell mass asymmetry is defined as: Δ*m* = 2*|*m_d1_*-*m_d2_*|/(*m_d1_*+*m_d2_*), where *m_d1_* and *m_d2_* are the masses of daughter cells 1 and 2, respectively, although we note that the daughter cell number does not play a role in the analysis output. Shape factor is defined as 4*pi*A/*P* ^2^, where *A* is projected area of a cell in pixels and *P* is the number of pixels around the boundary of a cell, as computed by the Matlab ‘region props’ function. Shape factor is a measure of cell roundness, with area determined based on image segmentation, as described above. The time the parent cell spent in mitosis is defined as the time from the midpoint of this abrupt increase in mass per area until the cell split into two daughter cells, as determined by image segmentation based on the watershed transform, a time point corresponding to the presence of a deep cleavage furrow.

Cell cycle synchronized mass asymmetry was determined by computing the coefficient of variance (CV  =  standard deviation/mean) of population mass vs. time data for all control, untreated L cells binned into 10 minute intervals from *t* = −90 min (before division) to *t* = 90 min (after division).

### Statistics

Statistical analysis between the experimentally determined means of individual treatment groups relative to control (Fig. 4, S4B Fig.) was performed using a two-tailed Welch's Student *t* test with unequal variances and sample sizes [34]. The slope of growth rate versus time and mass (S2B and C Fig.) and daughter cell Δ*m* versus confluence and experiment time (S3B and C Fig.) were analyzed using an F-test of the least squares linear regression in Matlab (Mathworks), testing the null hypothesis that the linear fit model does no better at describing the growth rate versus time/mass than a single constant term [34].

## Discussion

Our results show ∼10% mass asymmetry throughout cytokinesis even in untreated cell lines, suggesting that mass partitioning is not dynamically regulated during cytokinesis. Treatment with a panel of drugs which disrupt the initial partitioning of mass resulted in the persistence of asymmetry throughout cytokinesis. In most cases, daughter cell asymmetry continues to increase after the daughter cells separate and reattach to the substrate, as the larger daughter cell increases in mass more quickly than the smaller daughter cell. This suggests the existence of other cell size regulatory mechanisms to ensure the maintenance of a consistently sized cell population over time [12]. Recent work on mammalian growth and mass accumulation regulation during the cell cycle which showed that growth rate in early G1 increased with cell size, with apparent growth rate regulation at the G1/S transition [14]. The observed redistribution of mass from one daughter cell to the other may be a consequence of external mechanical interactions. Mechanical cues are known to drive cell shape and mass partitioning during division [4], [35], and an asymmetric extracellular matrix (ECM) is known to influence cell polarity during division [36]. We hypothesize that random substrate inhomogeneity may play a role in the observed redistribution of mass between daughter cells.

Additionally, our results suggest that cytoskeleton integrity is essential for symmetric mass partitioning during cell division. In particular, these data are consistent with a model in which initial cell biomass partitioning is determined by microtubule and actomyosin activity based on initial spindle positioning [4], [27], [29]. Two different actin disrupting drugs, latrunculin A and cytochalasin B, provide evidence showing that blocking new actin scaffolds is sufficient for initial asymmetry, but that depolymerization of actin, as occurs with latrunculin A, is required for the persistence of mass asymmetries over time.

We also show that specific disruption of cytoskeletal activity using small molecule inhibitors leads to a marked increase in asymmetric mass partitioning between daughter cells. Many cellular factors that control mitotic spindle positioning are known tumor suppressors or oncoproteins [37], disruption of which could also lead to division asymmetries.

There is evidence of asymmetric partitioning of proteins targeted for degradation during cell division in mammalian cells [9], and recent work in yeast suggests that, in response to heat or oxidative stress, protein aggregates are asymmetrically partitioned between daughters, to prevent the aging of at least one daughter cell and its progeny [38], [39]. Future work should examine the role that stress and asymmetric partitioning of protein aggregates plays in mass partitioning asymmetry, particularly in response to oxidative stress (e.g. phototoxicity).

Additional future work could examine the relationship between asymmetric cell biomass at division and asymmetric cell fates. Little is known about daughter cell size and its impact on asymmetric cell fate determination. However, asymmetric segregation of PAR proteins have been well characterized in determining polarity in development [7] and the segregation of fate determinants is crucial in stem cell self-renewal versus differentiation [5]. Interestingly, the cytoskeleton also plays a large role in cell fate determination [40]. Our work suggests that inhibition of cytoskeletal remodeling may lead to larger biomass asymmetries with cell division, perhaps leading to distinct daughter cell fates.

## Supporting Information

S1 Fig **LCI data from a single dividing L cell pair.** (A) mass versus time. (B) mean phase shift of the phase signal versus time. (inset) sigmoid filter (red) used to detect cell divisions. Sigmoid filter was moved along the phase signal until the best fit location was identified, in this case, *t* = −19 min, and the sigmoid function is a good fit to the data, indicating mitotic entry. An inverted sigmoid shape was used to detect mitotic exit of daughter cells. (C) normalized difference between mass of daughter cells versus time after division. (D) heatmap of daughter cell difference versus time, as in Fig. 3A.
(TIF)

S2 Fig **Averaged relative mass and mean phase shift data for control (untreated) mouse L cells.** (A) relative mass versus time with grey line shows the average value within each 60 minute bin. (B) binned growth rate (rate of change of mass over time) versus relative mass. (C) binned growth rate versus time. Grey line in (B) and (C) represents a linear fit to the growth rate data. The slope of these lines is positive with *p*<10^−4^, for data in both B and C. (D) mean phase shift versus time, with grey line showing the average value within each 6 minute bin. Error bars represent s.d. For clarity, only one out of every four error bars is shown in panel D.
(TIF)

S3 Fig **Effect of culture confluence on cell division asymmetry.** (A) distribution of cell confluence for all measured L cell imaging locations. Confluence is defined as the percent area covered by cells in the first frame of imaging. (B) daughter cell Δ*m* versus initial confluence at the daughter cell's imaging location for 1276 control L cells. Red line shows average Δ*m* binned based on 2 percentage point wide confluence bins. (C) daughter cell Δ*m* versus time of division (relative to the start of imaging for 1276 control L cells. Red line shows average Δ*m* binned into 4 hour wide bins. The slope of the best fit lines to daughter cell Δ*m* versus confluence (B) and daughter cell Δ*m* versus time (C) are not statistically significant, indicating no effect. Error bars show +.- s.e.m.
(TIF)

S4 Fig **Cell tracking results from drug treatment experiments.** (A) number of tracked divisions per hour of observation at each imaging location for each condition studied. (B) measured specific growth rate (growth rate divided by mass) at each experimental condition. (C) average daughter cell Δ*m* versus experiment time for baseline concentration drug treatments binned into 4 hour wide bins. Control, C (*n* = 542); DMSO, D, 0.06% (*n* = 734); latrunculin A, L, 100 nM (number of low concentration cells, *n_lo_*, = 79, number of baseline concentration cells, *n_baseline_*, = 162, number of high concentration cells, *n_hi_*, = 21); blebbistatin, B, 10 µM (*n_lo_* = 68, *n_baseline_* = 156, *n_hi_* = 55); nocodazole, N, 75 nM (*n_lo_* = 125, *n_baseline_* = 136, *n_hi_* = 3); cytochalasin B, Y, 2 µM (*n_lo_* = 105, *n_baseline_* = 101, *n_hi_* = 11). Low (lo) and high (hi) concentrations are 1/2x and 2x the baseline concentration, respectively. Red lines in A indicate the division rate for individual experiments. Error bars in A show the estimated standard error based on the poisson distribution  =  square root of number of events scaled by the number of hours of observation, which in most cases shows a lower variation than the measured division rate of individual experiments. Error bars in B and C represent s.e.m. over the number of experimental replicates. * *p*<0.05.
(TIF)

S5 Fig **Shape factor dynamics during cell division with cytoskeletal inhibitors.** (A) daughter cell asymmetry and shape factor at *t* = 10 min (solid lines) and *t* = 50 min after division (dashed lines). (B) Average shape factor versus time after division shows a steady decrease as cells reattach to the substrate and lose their round shape following division. Error bars represent s.e.m.
(TIF)

S1 Movie **Movie of cell division shown in** **Fig. 2A** **.** Movie of symmetrically dividing mouse L cell. Red outline shows the identified outline of cell by the watershed transform. The dividing pair (data for which is shown in Fig. 2A) is centered in the movie frame.
(MP4)

S2 Movie **Movie of cell division shown in** **Figure 2C** **.** Movie of asymmetrically dividing mouse L cell. Red outline shows the identified outline of cell by the watershed transform. The dividing pair (data for which is shown in Fig. 2C) is centered in the movie frame.
(MP4)

S3 Movie **Movie of cell division shown in** **Fig. 2E** **.** Movie of dividing L cell showing change from asymmetric to symmetric mass partitioning. Red outline shows the identified outline of cell by the watershed transform. The dividing pair (data for which is shown in Fig. 2E) is centered in the movie frame.
(MP4)

S4 Movie **Movie of cell division shown in** **Fig. 2G** **.** Movie of dividing L cell showing change from symmetric to asymmetric mass partitioning. Red outline shows the identified outline of cell by the watershed transform. The dividing pair (data for which is shown in Fig. 2G) is centered in the movie frame.
(MP4)
