# Supplementary figures and images for: Live Cell Interferometry Quantifies Dynamics of Biomass Partitioning during Cytokinesis
Source: PLoS One. 2014 Dec 22;9(12):e115726. doi: 10.1371/journal.pone.0115726 (PMC4274116; doi:10.1371/journal.pone.0115726)

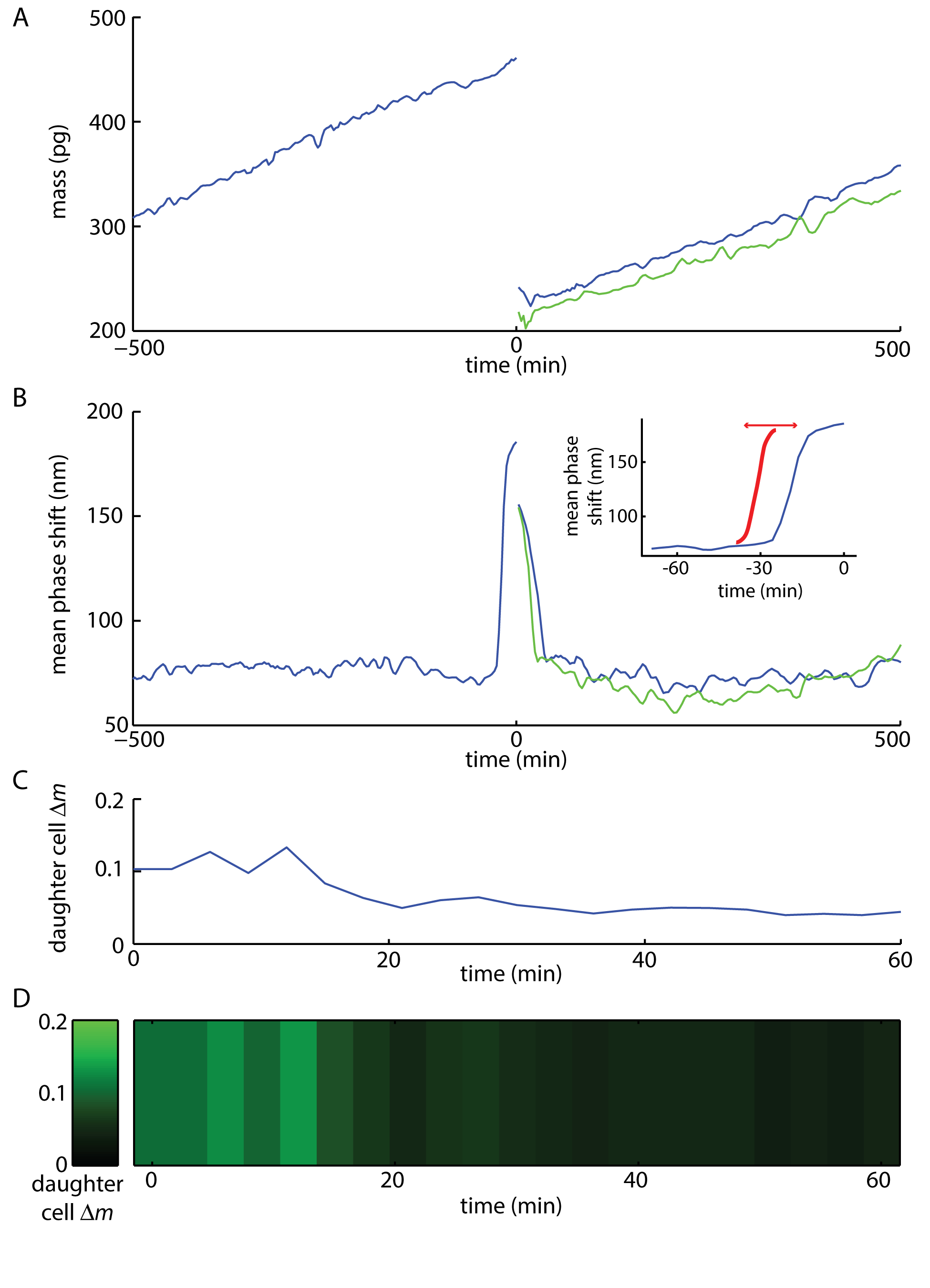

Supplement: S1 Fig — LCI data from a single dividing L cell pair. (A) mass versus time. (B) mean phase shift of the phase signal versus time. (inset) sigmoid filter (red) used to detect cell divisions. Sigmoid filter was moved along the phase signal until the best fit location was identified, in this case, t = −19 min, and the sigmoid function is a good fit to the data, indicating mitotic entry. An inverted sigmoid shape was used to detect mitotic exit of daughter cells. (C) normalized difference between mass of daughter cells versus time after division. (D) heatmap of daughter cell difference versus time, as in Fig. 3A. (TIF) [file pone.0115726.s001.tif]

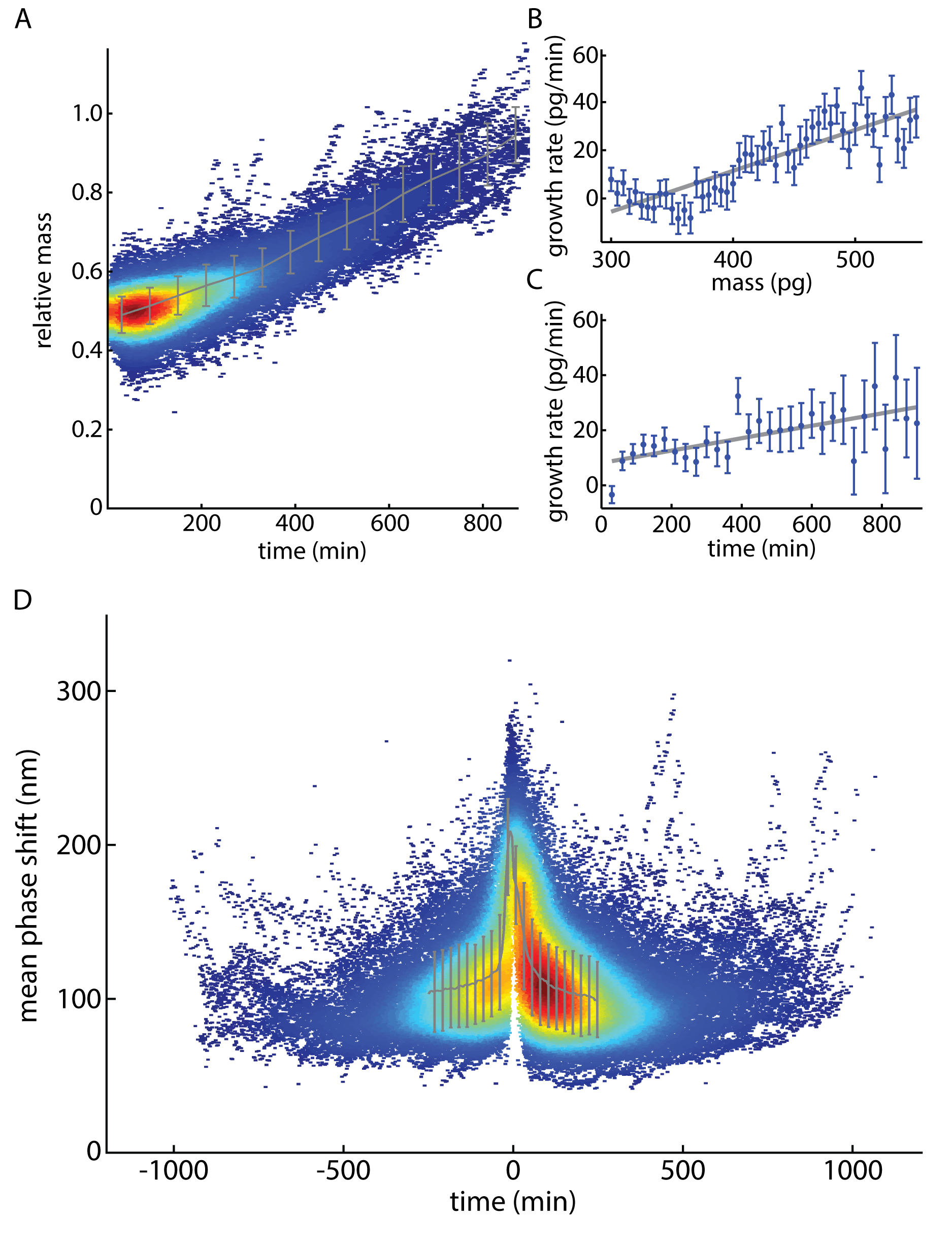

Supplement: S2 Fig — Averaged relative mass and mean phase shift data for control (untreated) mouse L cells. (A) relative mass versus time with grey line shows the average value within each 60 minute bin. (B) binned growth rate (rate of change of mass over time) versus relative mass. (C) binned growth rate versus time. Grey line in (B) and (C) represents a linear fit to the growth rate data. The slope of these lines is positive with p<10−4, for data in both B and C. (D) mean phase shift versus time, with grey line showing the average value within each 6 minute bin. Error bars represent s.d. For clarity, only one out of every four error bars is shown in panel D. (TIF) [file pone.0115726.s002.tif]

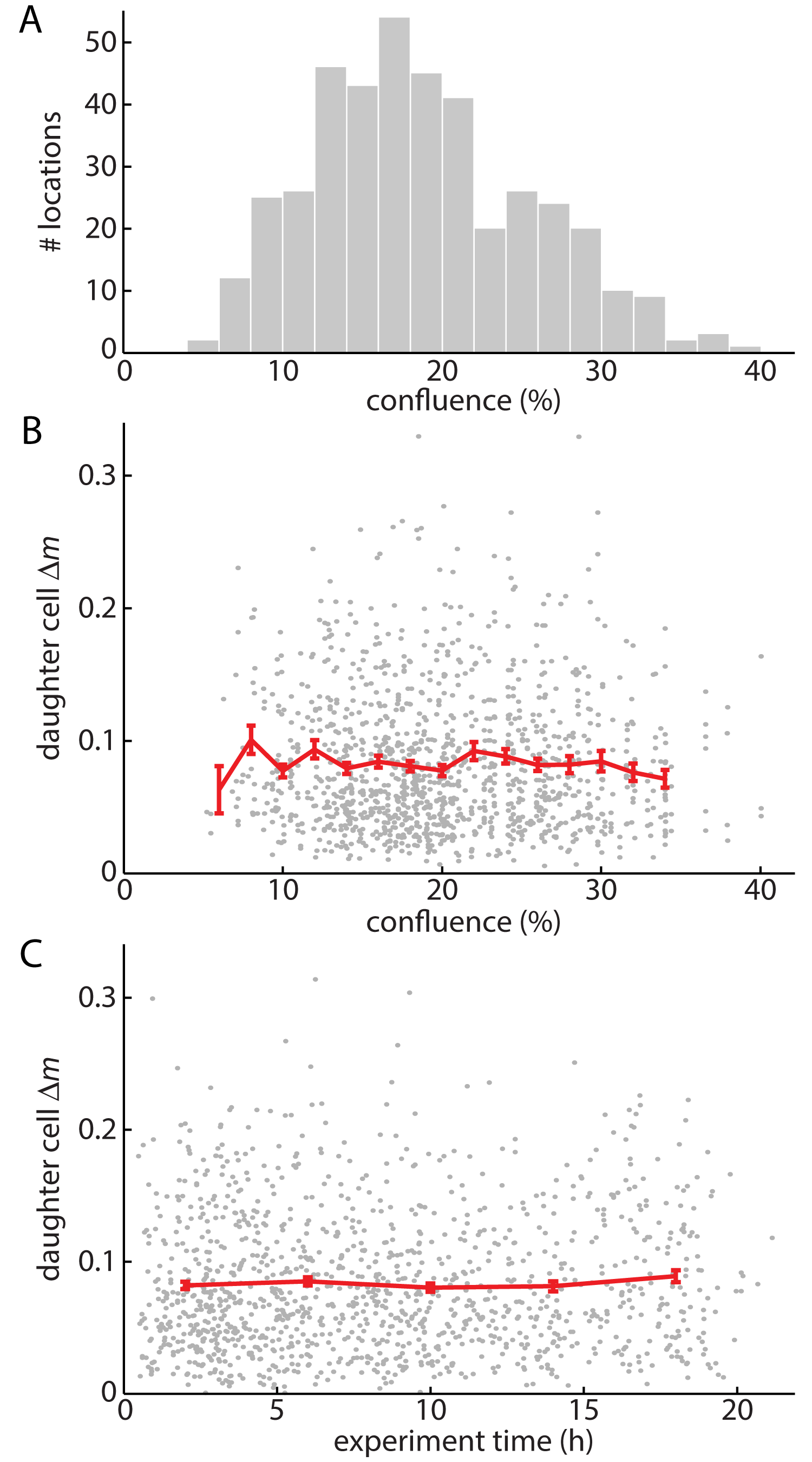

Supplement: S3 Fig — Effect of culture confluence on cell division asymmetry. (A) distribution of cell confluence for all measured L cell imaging locations. Confluence is defined as the percent area covered by cells in the first frame of imaging. (B) daughter cell Δm versus initial confluence at the daughter cell's imaging location for 1276 control L cells. Red line shows average Δm binned based on 2 percentage point wide confluence bins. (C) daughter cell Δm versus time of division (relative to the start of imaging for 1276 control L cells. Red line shows average Δm binned into 4 hour wide bins. The slope of the best fit lines to daughter cell Δm versus confluence (B) and daughter cell Δm versus time (C) are not statistically significant, indicating no effect. Error bars show +.- s.e.m. (TIF) [file pone.0115726.s003.tif]

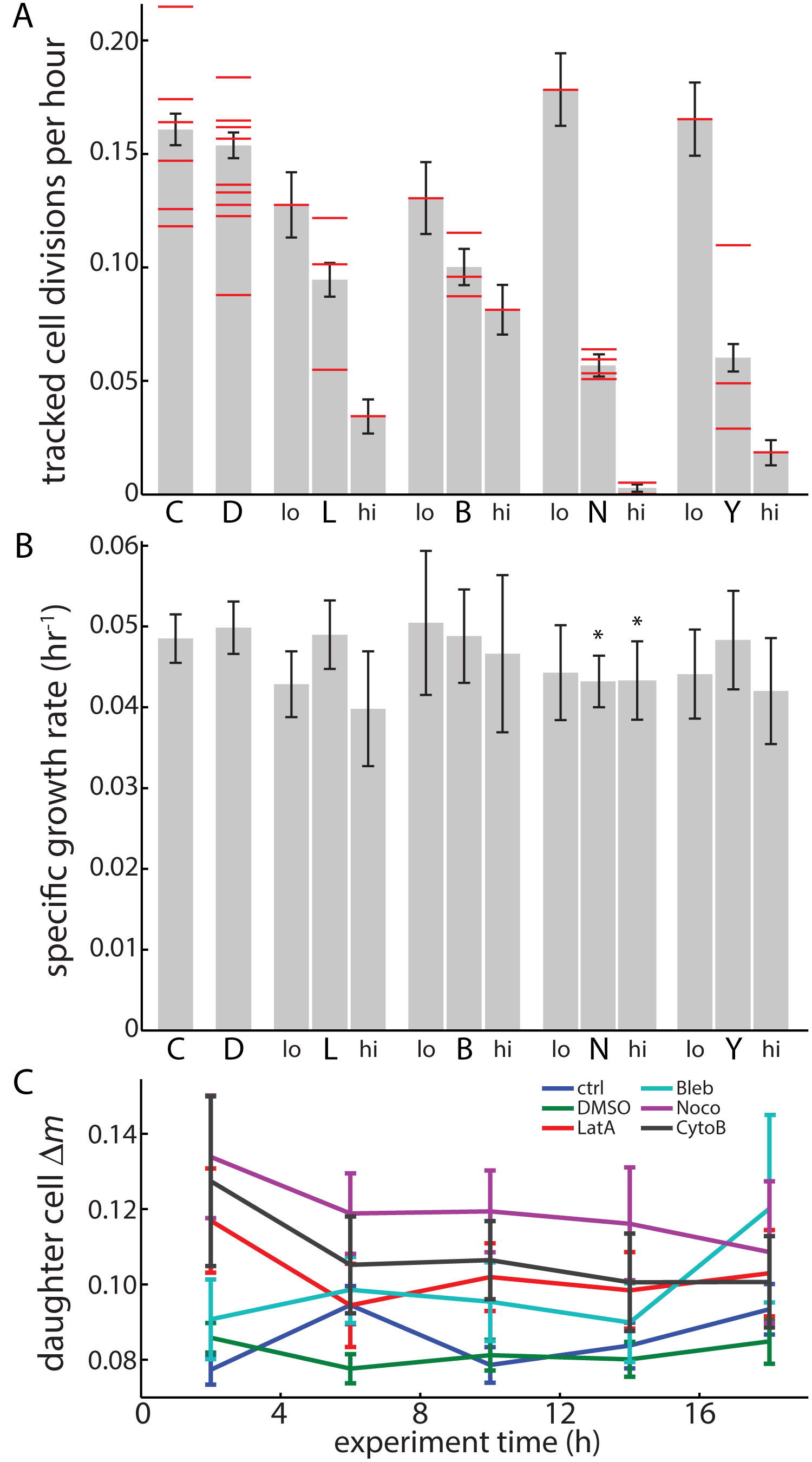

Supplement: S4 Fig — Cell tracking results from drug treatment experiments. (A) number of tracked divisions per hour of observation at each imaging location for each condition studied. (B) measured specific growth rate (growth rate divided by mass) at each experimental condition. (C) average daughter cell Δm versus experiment time for baseline concentration drug treatments binned into 4 hour wide bins. Control, C (n = 542); DMSO, D, 0.06% (n = 734); latrunculin A, L, 100 nM (number of low concentration cells, nlo, = 79, number of baseline concentration cells, nbaseline, = 162, number of high concentration cells, nhi, = 21); blebbistatin, B, 10 µM (nlo = 68, nbaseline = 156, nhi = 55); nocodazole, N, 75 nM (nlo = 125, nbaseline = 136, nhi = 3); cytochalasin B, Y, 2 µM (nlo = 105, nbaseline = 101, nhi = 11). Low (lo) and high (hi) concentrations are 1/2x and 2x the baseline concentration, respectively. Red lines in A indicate the division rate for individual experiments. Error bars in A show the estimated standard error based on the poisson distribution = square root of number of events scaled by the number of hours of observation, which in most cases shows a lower variation than the measured division rate of individual experiments. Error bars in B and C represent s.e.m. over the number of experimental replicates. * p<0.05. (TIF) [file pone.0115726.s004.tif]

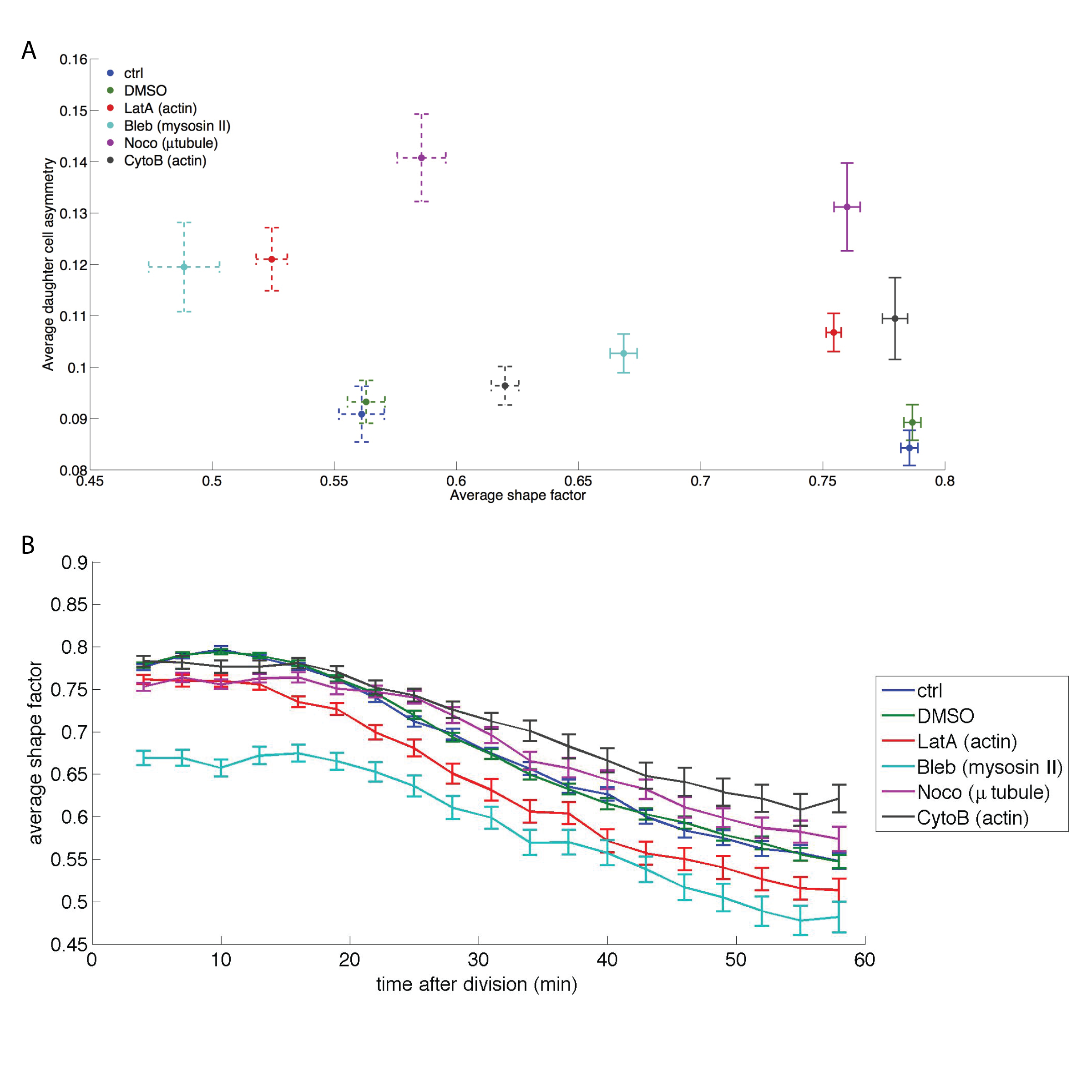

Supplement: S5 Fig — Shape factor dynamics during cell division with cytoskeletal inhibitors. (A) daughter cell asymmetry and shape factor at t = 10 min (solid lines) and t = 50 min after division (dashed lines). (B) Average shape factor versus time after division shows a steady decrease as cells reattach to the substrate and lose their round shape following division. Error bars represent s.e.m. (TIF) [file pone.0115726.s005.tif]
